# Observing spontaneous, accelerated substrate binding in molecular dynamics simulations of glutamate transporters

**DOI:** 10.1371/journal.pone.0250635

**Published:** 2021-04-23

**Authors:** Jiali Wang, Peifan Li, Xiaozhen Yu, Christof Grewer

**Affiliations:** Department of Chemistry, Binghamton University, Binghamton, New York, United States of America; Universidade de Sao Paulo Instituto de Quimica, BRAZIL

## Abstract

Glutamate transporters are essential for removing the neurotransmitter glutamate from the synaptic cleft. Glutamate transport across the membrane is associated with elevator-like structural changes of the transport domain. These structural changes require initial binding of the organic substrate to the transporter. Studying the binding pathway of ligands to their protein binding sites using molecular dynamics (MD) simulations requires micro-second level simulation times. Here, we used three methods to accelerate aspartate binding to the glutamate transporter homologue Glt_*ph*_ and to investigate the binding pathway. 1) Two methods using user-defined forces to prevent the substrate from diffusing too far from the binding site. 2) Conventional MD simulations using very high substrate concentrations in the 0.1 M range. The final, substrate bound states from these methods are comparable to the binding pose observed in crystallographic studies, although they show more flexibility in the side chain carboxylate function. We also captured an intermediate on the binding pathway, where conserved residues D390 and D394 stabilize the aspartate molecule. Finally, we investigated glutamate binding to the mammalian glutamate transporter, excitatory amino acid transporter 1 (EAAT1), for which a crystal structure is known, but not in the glutamate-bound state. Overall, the results obtained in this study reveal new insights into the pathway of substrate binding to glutamate transporters, highlighting intermediates on the binding pathway and flexible conformational states of the side chain, which most likely become locked in once the hairpin loop 2 closes to occlude the substrate.

## Introduction

Excitatory amino acid transporters (EAATs), which belong to the solute carrier 1 (SLC1) family, are an important class of membrane proteins expressed in the mammalian central nervous system, where they transport glutamate across neuronal and astrocytic membranes. EAATs take up glutamate into cells against a large concentration gradient [1, 2], which prevents the glutamate concentration from reaching neurotoxic levels in the extracellular space [2, 3]. The dysfunction of glutamate transporters is proposed to be involved in several diseases, including stroke and Alzheimer’s disease [4, 5]. However, the exact involvement of EAATs in these diseases is not fully understood.

Glutamate transporters transfer Na^+^, H^+^ and glutamate from the extracellular to the intercellular side of membrane, in exchange for one potassium ion in the reverse direction (stoichiometry of 3:1:1:1), resulting in a net total of two positive charges moving from the outside to the inside of the cell [1, 2, 6]. These ion fluxes are coupled to conformational changes of the transporter, leading to the alternating access of the glutamate and cation binding sites.

Direct structural evidence for the mechanism of substrate translocation was first provided by two crystal structures of the archaeal glutamate transporter homologue Glt_*ph*_ [7, 8]. In these structures, outward-facing (OF) and inward-facing (IF) states were observed. Glt_*ph*_ shares the same fold and more than 30% sequence identity with mammalian glutamate transporters. However, more recently, structures of the human glutamate transporter EAAT1 (excitatory amino acid transporter 1) were published [9], providing further important information for structure, function, and molecular dynamics simulation studies.

The current understanding of the structural changes involved in substrate translocation is based on the “elevator-like” [10–12] alternating access transport mechanism. Here, the structure starts with the “outward-occluded” state of Glt_*ph*_, in which bound substrate is occluded by a closed external gate, presumably hairpin loop 2 (HP2) and the aspartate binding site is not accessible from the extracellular solution [13]. A large, elevator-like movement of the transporter domain along the membrane normal [14] brings the substrate binding site closer to the intracellular solution, transforming the transporter into the inward-facing (IF) state [7]. During this process, the bound substrate is translocated through the lipid bilayer.

Previous studies have indicated critical roles of amino acid residues in the two hairpin loops, HP1 and HP2 [15–18], located at the center of the transport domain, which also includes transmembrane domains 7 and 8 (TM7, TM8). The HP2 loop undergoes large scale conformational changes to control the exposure of the substrate binding region to the extracellular solution [15–17, 19]. Therefore, HP2 has been proposed to act as a gate [17] to close off the substrate binding site from the aqueous environment. Aspartate binding was proposed to occur through an induced fit mechanism, with loop closure following aspartate association [20, 21]. Also, cations binding at the Na2 sodium binding site largely enhance the propensity for a closed-gate conformation [18, 19, 22, 23].

In addition to the glutamate translocation process [14, 24], the structural mechanism of substrate interaction with the binding site, including time-resolved mechanisms of binding and dissociation, have been investigated in molecular dynamics (MD) simulation studies [16, 25–28]. The successive steps of gate opening and closing, substrate recognition and eventual binding occur in nanoseconds [18]. However, observation of spontaneous substrate binding in MD simulations is a time-consuming process, requiring long-duration simulation runs. The random walk nature of substrate diffusion in solution and the lipid environment increase the simulation time.

In this work, we apply and compare three methods that accelerate MD simulations of the substrate binding process [29] to study the substrate-transporter successive interaction steps during association reaction. These methods reduced a microseconds simulation under conventional molecular dynamics to the 10–100 nanosecond-level. The results suggest a potential aspartate-bound intermediate state from trajectory analysis. At the intermediate state, the substrate could bind and demonstrate a residency time of more than 50 ns. In addition, the results indicate that the side chain carboxylate group retains significant conformational flexibility in the initial binding state, before HP2 closes. The simulations were also extended to glutamate binding to EAAT1, for which a crystal structure is not known in the glutamate-bound state.

## Results

### Observing accelerated, spontaneous substrate binding by adding user-defined force

**Fig 1:**
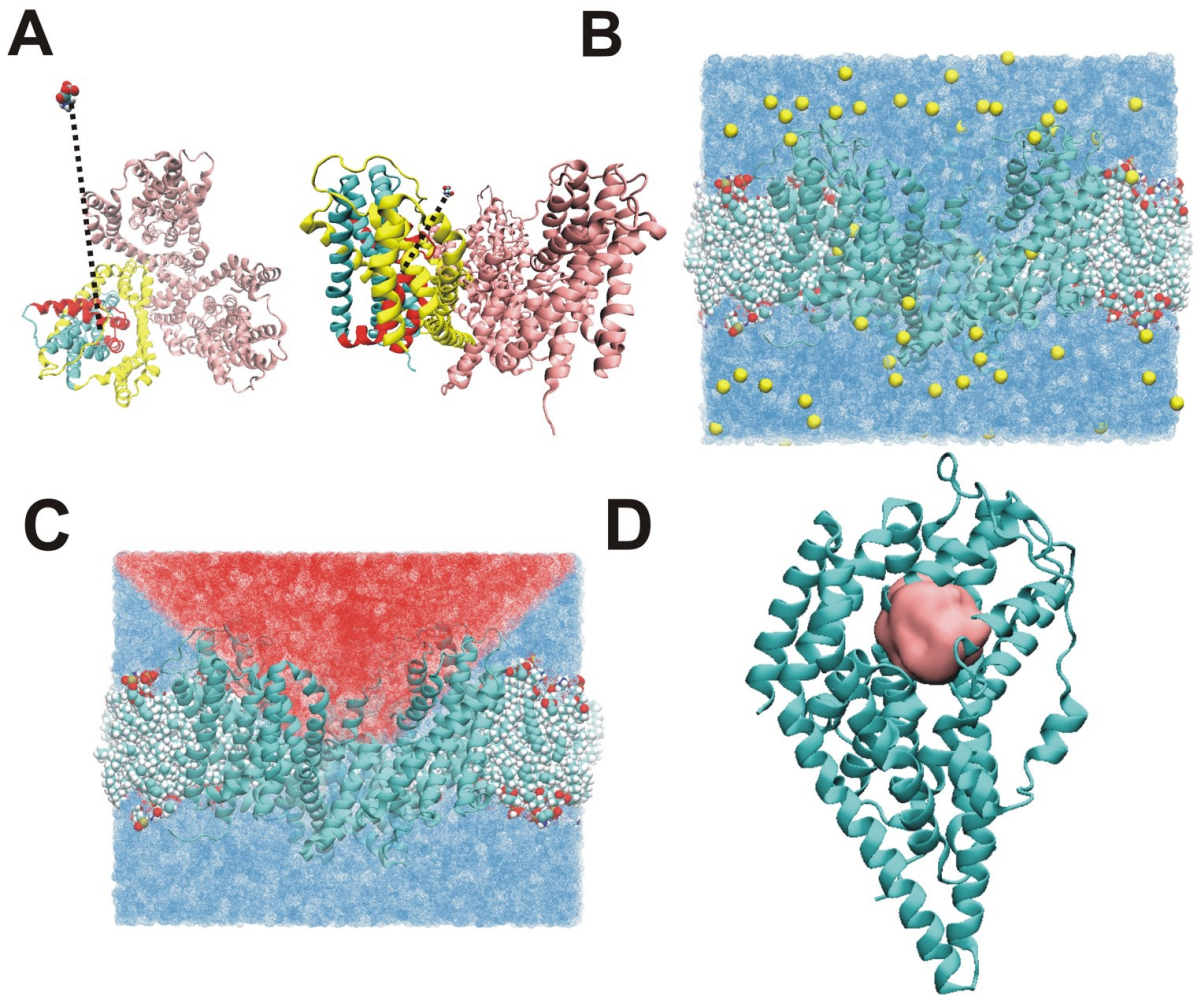
Simulation environment and region of force application. The initial structure of the transporter in the simulation displayed as top-view and side-view are shown in (A). A typical initial position of the aspartate molecule and its relative distance to the binding site is illustrated by the dashed line. (B) Setup of the simulation environment, Glt_ph_ structure (2nwx) with three subunits (cyan). Only Na1- and Na3-bound state were used to insert into the lipid bilayer (drawn in VDW representation) and the simulation environment was generated and illustrated in (B). The yellow spheres indicate sodium ions at 0.15 M concentration. The blue color in the background shows water molecular drawn using solvent visualization in VMD. Lipids are shown in VDW mode and all pictures were generated using VMD. We used *x*^*2*^*+y*^*2*^*<(z+15)* and *z>0* Å to define the selection region (red) for external force application. The selection region is illustrated in a side-view (C). (D) Force cut-off region (pink) within 7 Å of reference atom (Arg-397, CZ). Details of region selection are outlined in ***Materials and Methods***.

MD simulations using the Glt_*ph*_ structure were sampled after generating a lipid bilayer-solvent environment with a dimension of 120*120*100 Å^3^. The system contained 0.15M NaCl and was neutralized (Fig 1A and 1B). Simulations were run using the CHARMM36 [30, 31] and CHARMM27 [32] force fields. In the initial simulations, described in the next paragraphs, both the Na1 and Na3 sites were occupied, while the Na2 site was empty. In order to be able to observe the substrate binding process with reasonable simulation times, we used three methods, resulting in apparent acceleration of the substrate binding process. The first method is based on a user-defined force protocol (S1 Fig) and was used with NAMD 2.13 software. The method reduces unnecessary simulation steps when substrate is far away from the protein or target residues (Fig 1A), with which the substrate is known to interact within the binding site. The external force with constant strength added to the simulation system aims to push substrate toward its target and accelerate the binding process, when the substrate is at a large, user-defined distance from the binding site (Fig 1B–1D). However, when the substrate is close enough to the target residues (at a cutoff distance that can be adjusted in the protocol), the external force is turned off, which makes studies of accelerated spontaneous binding possible (S1 Fig).

The simulation was first initiated with aspartate at a randomly-chosen position in the extracellular solution. The initial structures for the simulations were generated after 5–10 ns equilibration and production runs using the Glt_*ph*_ structure (Materials and methods). After 5–10 ns simulation, the HP2 partial open state (Fig 1A) is formed, typically within one or two subunits, in agreement with previous reports [15, 17, 18]. Because of periodic boundary conditions of the simulation box, user-defined force has no physical meaning when the substrate moves across the periodic boundary to the intracellular side of the membrane. Therefore, we first define a cone as the selection region for force application (Fig 1C and 1D) and only apply external force in extracellular part of the simulation box when the substrate moves into this region (S1 Fig).

User-defined force calculations were started after forming the HP2 open state (Fig 1A), and after placing free aspartate at two different positions (23 Å or 18 Å from the reference residue), with similar results. The flowchart of the calculation protocol is illustrated in S1 and S2 Figs. In the flow control part, we use *x*^*2*^*+y*^*2*^*<(z+15)* and *z>0* Å to define the cone region as the first decision criterion. The second decision was made to either execute the *addforce* option or to go to conventional MD (no force application). Here we use dx = 7 Å to define the boundary as the cut off distance, illustrated as the sphere in Fig 1, and use Arg-397, which is known to coordinate the β-carboxylate of aspartate [33], as the reference residue to track the distance change. At this distance, it is expected that the substrate should not form specific intermolecular interactions with the binding site, except for long-range electrostatic forces (cut-off set to 12 Å, see Materials and methods). In six 30–80 ns MD simulations, aspartate was observed to spontaneously bind to the target residue.

**Fig 2:**
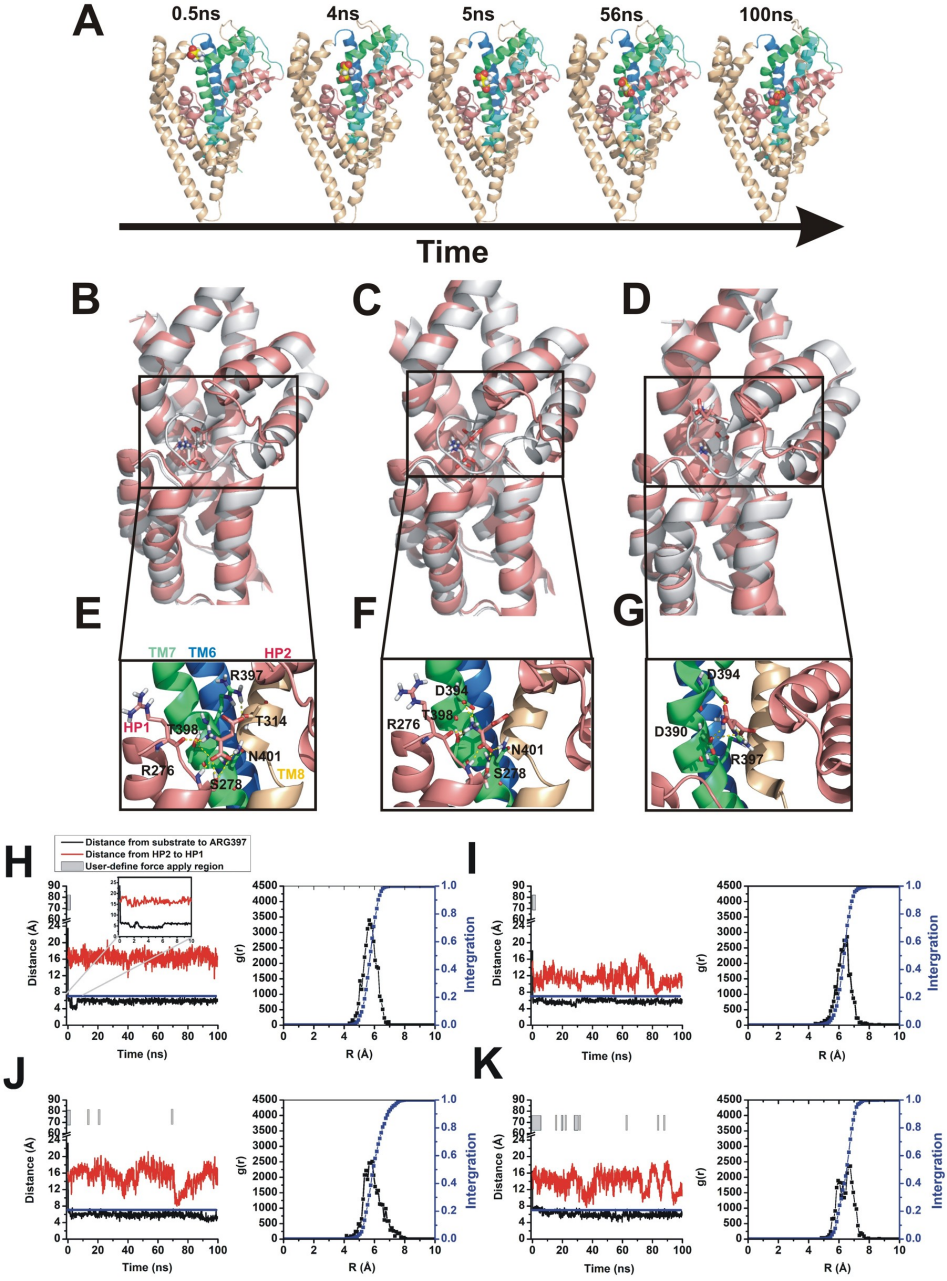
HP2 gate function and aspartate coordination in user-defined force simulation. (A) Substrate-protein successive binding steps. In simulations with user-defined force, within a short period of simulation time, aspartate will move close to HP2 loop, but the binding pocket is not accessible when the HP2 loop is not fully open. Once the open-gate state forms (HP2-HP1 distance larger than 12 Å), aspartate is able to move inside the binding pocket. At this moment, the conserved residues located in TM7 stabilize the aspartate. (B-D) Coordination states with open loop from user-defined force simulation results are shown in pink. Comparison of the crystal structure (2nwx) is shown in grey. All simulation results were from sampling by adding user-defined force to conventional MD simulations. Two distinguishable binding positions are shown in (D) and (E), (F). One overlaps with the binding site in the crystal structure (D) and another (E, F) is located at a higher Z-axis position. (E) Aspartate bound in HP2 half-open state, and (F, G) in HP2 fully-open state. (H-K) Time evolution of distance calculation (black) and HP2-HP1 distance (red) shown in each picture, respectively. The blue line indicates the user-defined force boundary and the grey bar region indicates the time of force application. In the region below the blue line (distance<7Å) or not covered by the grey bar, no user-defined forces were added to substrate, and conventional MD were sampled here. The right panels of each graph show aspartate radial distribution functions (black) and cumulative radial distribution functions (blue). Results were analyzed within 10 Å distance from the reference Arg-397 residue (atom CG).

In the trajectory illustrated in Fig 2A, in which the HP2 loop is initially only partially open, aspartate moves close to the binding pocket but does not bind, as access to the binding site is controlled by HP2. The degree of opening of HP2 can be assessed by the distance between the tips of HP1 and HP2. At the starting point of the simulation, the HP2-HP1 distance was around 8 Å, which indicates a partial-open state (the distance in the aspartate-bound, occluded state is 5.6 Å and in the TBOA-bound state, in which HP2 is propped open by the inhibitor, is 12.6 Å).

Once HP2 adopted the fully open form (distance between HP1 and HP2 of 12 Å, for simulations that show the time dependence of opening of HP2 after aspartate removal from the binding site see S3 Fig) aspartate was able to access the binding site within 10 ns (Fig 2A). From analysis of the trajectories of the simulations, three relevant conformations were observed on the binding pathway, shown in Fig 2B–2D). From left to right: Aspartate binding in the original binding pocket (Fig 2B and 2C), binding at an intermediate site with partial-open loop (Fig 2D). The first conformation compares very well with the binding position and coordination of aspartate in the Glt_*ph*_ crystal structure [34, 35], suggesting that the accelerated binding protocol can provide meaningful results on the actual physical binding pose of the substrate. Asp-394 is known to coordinate the positively-charged amino group of aspartate, most likely through electrostatic interaction, however, an additional *syn-*conformation (see below) of the substrate was formed in the simulation, which could be an intermediate on the pathway to the stable configuration found in the crystal structure, pointing to flexibility of the ligand before stable binding is attained. The most stable state (crystal structure) needs the HP2 loop to close on the ligand, which forms additional H-bonds, involving residues A358 and V355 backbone oxygen atoms.

Details of a third coordination state of aspartate are shown in Fig 2E–2G. In this intermediate state, Asp-390 and Asp-394 contribute to binding of aspartate, Arg-397 located in TM8 participates in binding. In the binding position, the carbonyl oxygen from side chain of Asp-390, Asp-394, together with side chain of carboxamide group of Arg-397, contribute to aspartate binding. A magnified illustration of this intermediate state is shown in S4 Fig. This intermediate state was not observed in all simulations, so it is likely not necessary to be occupied during along the binding pathway. Fig 2B and 2C show a close-up comparison of the binding modes from the simulation results. It is evident that the position of the amino group from the simulation results overlaps with that in the crystal structure, but the two acidic carboxyl groups display deviating conformation. The conformation of aspartate here is reminiscent of a *syn-*conformation (dihedral angle about 70°), in which the aspartate side chain carboxylate and the α-carboxylic acid group face roughly the same side of the single bond. This conformation is different compared to the *anti-*conformation resolved by X-ray crystallography, in which the two carboxylate groups face away from each other (dihedral angle about 180°).

To more quantitatively analyze the results from the MD binding simulations, we evaluated aspartate binding trajectories (Fig 2H–2K, left). These trajectories show that after starting the simulations, user-defined force was only necessary for the first 2–10 ns of simulation time, after which the aspartate substrate spontaneously accessed the binding site and remained stable for 100s of ns. Similar behavior was observed in all three monomers. At the same time, the HP2 loop did not close, or closed only partially. This indicates that binding is a two-step process, with initial, rapid association with the HP2-open binding site, and subsequent slow closing of HP2 to form the outward-closed conformation, which is competent for translocation. This HP2 loop closure, which occurs on a longer time scale and involves binding of sodium to the Na2 site, was not observed in the simulations presented here.

The aspartate bound state on the binding pathway could be distinguished from radial distribution function (RDF) analysis, as shown in Fig 2H–2K (right). Population states were defined by a difference in distance from the substrate (CA atom) to Arg-397 (CZ atom), apparent as RDF peaks at 5–6.5 Å. In the crystal structure, this distance is around 5.6 Å in 2nwx (the corresponding distance in the TBOA bound structure is 5.5 Å).

In order to assess the Na^+^ effect on the simulations, we also generated a Glt_*ph*_ model without Na^+^ located in the Na1 binding sites (the Na3 site was still occupied, but the Na2 site was empty), but otherwise under the same simulation conditions described above. Aspartate was still able to spontaneously bind to the binding site, in binding poses illustrated in S5 Fig. However, once bound, aspartate was less stable and larger time of force application was needed to move aspartate close to the binding site. To quantify this analysis, S6 Fig shows a force-applied region spanning over 40% percent of the whole simulations time, whereas in the presence of Na^+^ in the Na1 site, less than 5% of the simulation time was spent in the force-applied region (S5A–S5C Fig), in Fig 2H–2K. Together with RDF plots show in S6 Fig, these results indicate a less stable environment for aspartate under non-occupied Na1 conditions near and in the binding site, as expected due to electrostatic compensation of the negative Na^+^ binding site by Na^+^ association [36].

Finally, we determined the effect of the cut-off distance for force application. For this purpose, we selected a larger cut-off distance of 10 Å, to compare with the results from the 7 Å cut-off simulations. In the sample trajectory shown in S7 Fig, force application was only needed in the first 5 ns, but the aspartate native binding pose only stabilized after about 50 ns. This stabilization process was much longer than in the simulations with a cut-off distance of 7 Å (within < 20 ns).

### Application of a flat-bottom potential to contain substrate near the binding site

**Fig 3:**
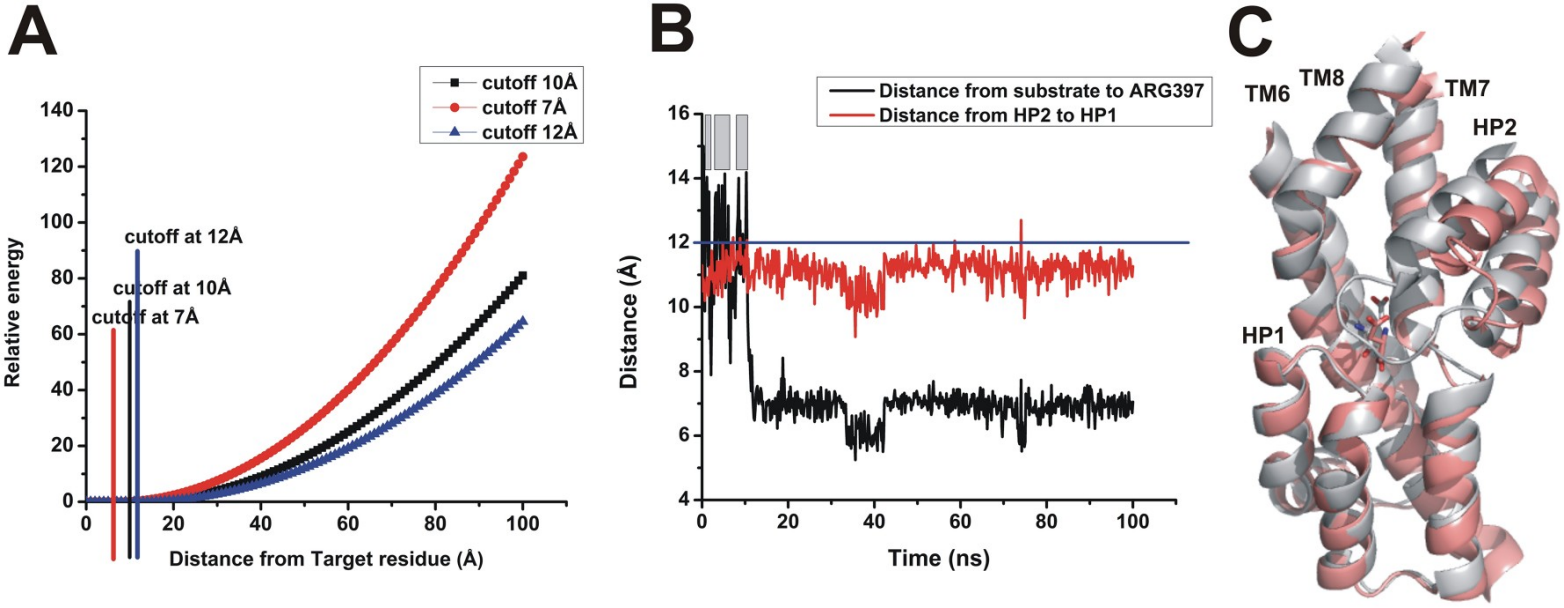
Flat-bottom harmonic potential to accelerate aspartate binding. (A) Illustration of the flat-bottom harmonic potential that was used with cutoff distances indicated in red (7 Å), black (10 Å) and blue (12 Å). (B) Typical trajectory for the aspartate binding process. The grey bars indicate the time spent beyond the zero energy cut-off distance. (C) Structural snapshot of the final state after 100 ns simulation time, compared with the Glt_*ph*_ crystal structure (2nwx).

Like in steered molecular dynamics (SMD), the application of a directional force, as described in the method used in the previous paragraphs, results in a non-equilibrium situation, in which irreversible work is performed on the simulation system. These non-equilibrium conditions could result in unknown effects on the observed binding pathway. Therefore, we repeated the accelerated simulations, but instead of using a user-defined force, aspartate was contained near the binding site by using a flat-bottom harmonic potential (illustrated in Fig 3A). Here, the potential was zero between the binding site target residue and a cutoff distance of 7–12 Å, after which the harmonic potential was applied.

Consistent with the results from the previous method, spontaneous aspartate binding was observed within the first 20 ns of the simulation, despite increasing the cutoff distance to 12 Å (Fig 3B). Once bound, aspartate was stable in the binding site for the remainder of the simulation. The final binding pose of aspartate at the end of a 100 ns simulation is shown in Fig 3C. Aspartate was initially bound in the *syn*-like conformation, although the *anti*-configuration, which was observed in the crystal structure, was also sampled during the trajectory. As in the results describe in the previous paragraphs, hairpin loop 2 did not close during the 100 ns simulation time.

### Spontaneous substrate binding using high aspartate concentrations

In order to further verify the results from the user-defined force and the flat-bottom potential simulations, a spontaneous binding process was investigated using a third method, namely conventional MD simulations (cMD). Here, high aspartate concentrations were created within the simulation cell. Under these condition, 76 free aspartate molecules were added manually to the aqueous solution in the simulation environment (final concentration ~0.1 M) at an even distribution throughout the extracellular water phase of the simulation box. High concentration environments are believed to accelerate the spontaneous binding process by increasing the binding probability, as has been previously demonstrated for identifying the Cl^-^ permeation pathway and potassium potential binding sites in glutamate transporters [24, 37]. Spontaneous binding simulations were run extending to three microseconds.

**Fig 4:**
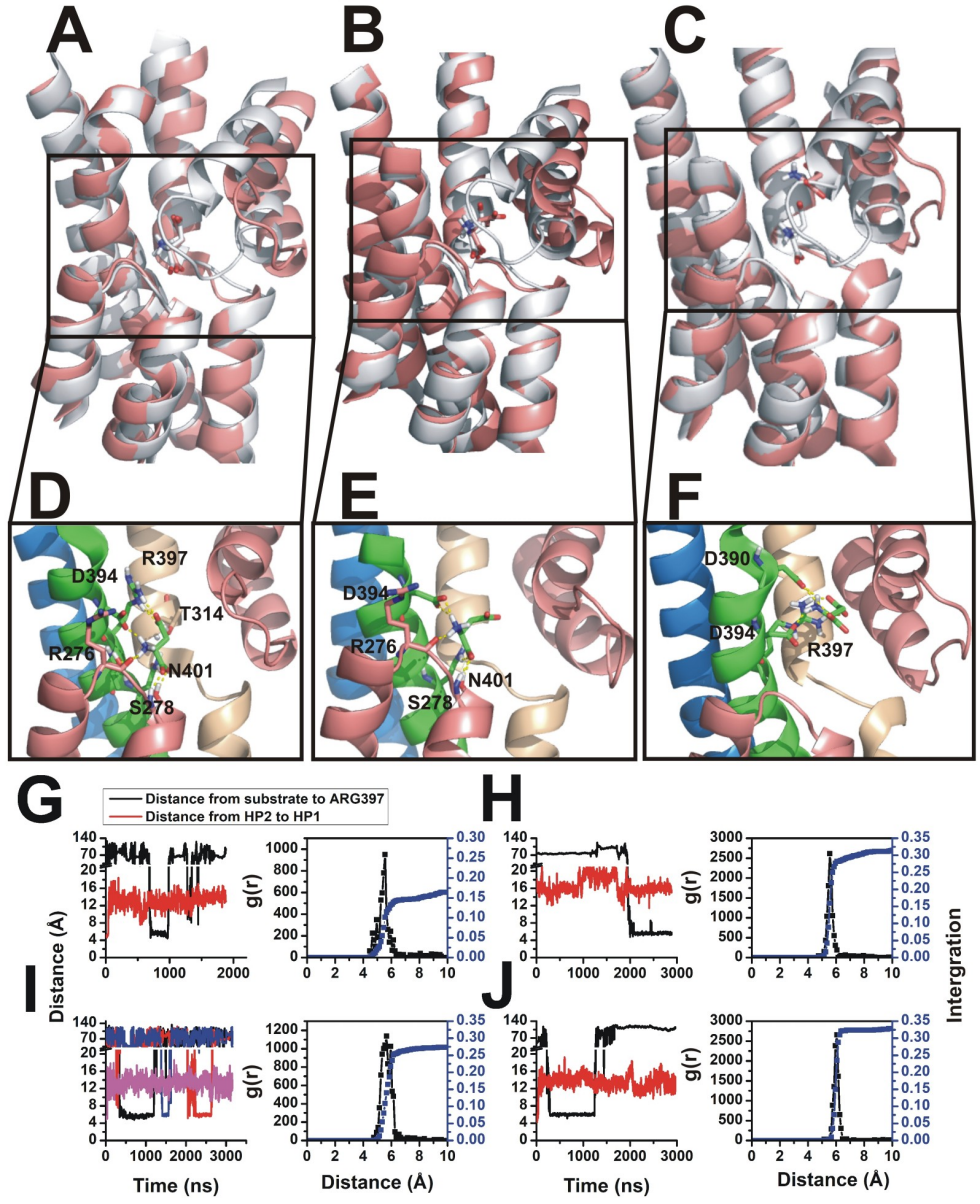
Coordination states during spontaneous binding to Glt_ph_ at high aspartate concentration. In MD spontaneous binding simulations at high aspartate concentration (~0.1 M), three major binding states are compared with the crystal structure were illustrate in (A) to (C). Aspartate coordination is shown magnified for each state in (D) to (E), respectively. The simulation results are illustrated in pink, and for comparison, the structure from the crystal structure (2nwx) is shown in grey. (A) and (B) are *syn* and *anti*-conformations of aspartate located in the binding pocket. (C) shows the intermediate state which binds to the same residues as in Fig 2D. Eight independent simulations were analyzed, and the time evolution results are shown from (G) to (J). Distance calculations are based on the same reference residues shown in Fig 2. Aspartate distance from the binding site reference residue (black) and HP2-HP1 distance (red) are shown in each panel. Radial distribution function analysis results are shown at the right of each trajectory. The different colors in (G) indicate different aspartate molecules binding and dissociating.

**Fig 5:**
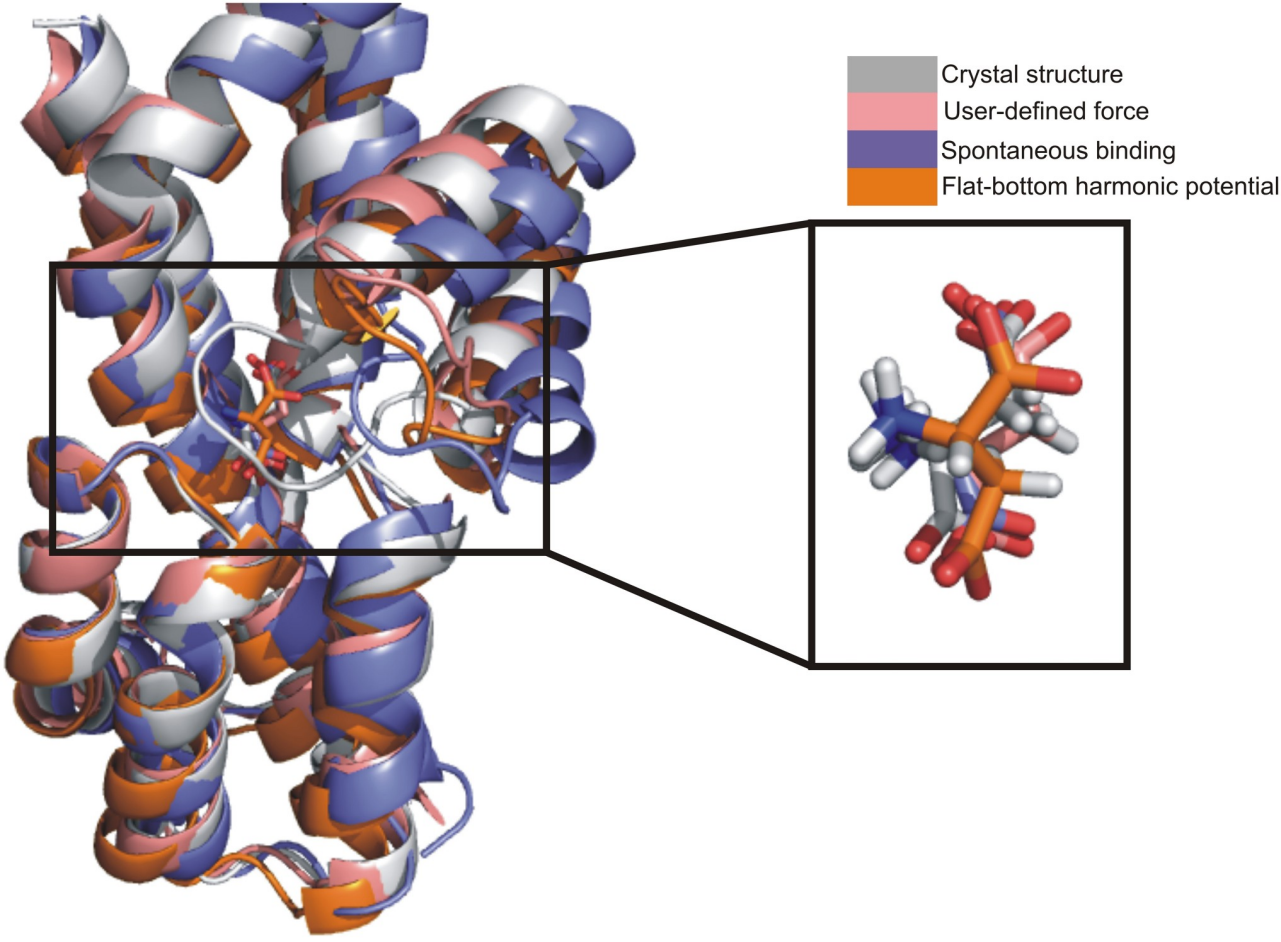
Comparison of four coordination states with aspartate located in the binding pocket. Three MD simulation methods in the *anti*-configuration are compared with the crystal structure (2nwx). Pink, purple and orange color protein structures are defined in the legend. The crystal structure (2nwx) is shown in grey.

Similar to the accelerated binding protocol described above, in the cMD simulation results aspartate was also observed in the binding pocket in both *anti*- (Fig 4A) and *syn*-conformations (Fig 4B). Snapshots were taken directly from the simulation trajectories. The same intermediate state shown in Fig 4C also occurred in cMD simulations, where the Asp-390 and Asp-394 residues contribute to aspartate stabilization (Fig 4D–4F). Distance analysis and radial distribution function results are shown in Fig 4G–4J. Similar results were obtained from data from eight independent simulations, in which simulation times ranged from 0.7 to 3 μs. Two stable states were found, one was the native pose in the original binding pocket (similar to x-ray structure, Fig 4D and 4E and the other one was a pose, in which Asp-390 also contributes to binding (Fig 4F). Overall, these results are very similar to those from the accelerated binding simulations. To further estimate the accuracy with user-defined method, we compared the aspartate binding states within binding pocket. As shown in Fig 5, three binding states were in different colors. The most populated *anti*-conformation was observed in all three methods. In Fig 5, right panel, we observe the backbone and side chain has only slightly differences in configuration when comparing between the three methods.

### Binding of glutamate to the mammalian glutamate transporter EAAT1

**Fig 6:**
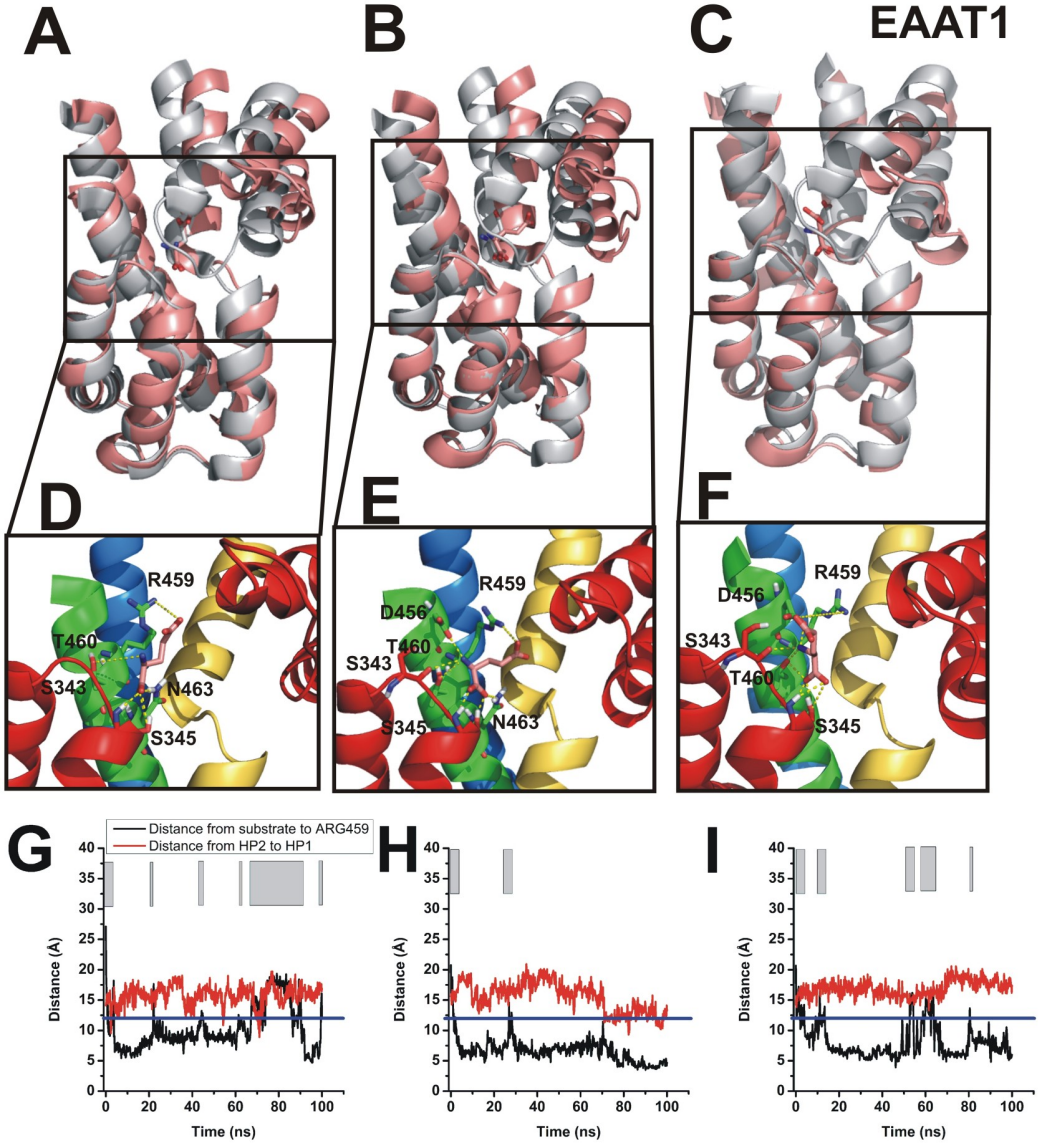
Glutamate binding to EAAT1 in flat-bottom harmonic potential accelerated simulations. (A-C) Representative conformations of glutamate after binding to EAAT1 using the same simulation protocol as shown in Fig 3 (flat-bottom potential, pink color). Comparison to the crystal structure (2nwx) is shown in grey. Magnification of glutamate coordination states are shown in (D-F). Time evolution of distance between glutamate and Arg 459 (black) and HP2-HP1 tip distance (red) (G-I). The blue line indicates the flat-bottom cut-off distance (distance < 12Å) and the grey bars indicate the time spent within the harmonic section of the potential.

While the crystal structure of the mammalian glutamate transporter EAAT1 is known in complex with aspartate, the glutamate-bound state has not been structurally characterized. Therefore, we tested whether it was possible to observe spontaneous glutamate binding to EAAT1. We used the second method, i.e. a flat-bottom harmonic potential to prevent diffusion of glutamate too far away from the binding pocket. The cutoff distance was set to 12 Å. As shown in Fig 6A–6C, glutamate binding was observed to all three subunits in the EAAT1 homo-trimer with binding poses that resemble aspartate binding for the α-carboxy and amino positions, but significantly higher variability in the side chain (see Fig 6D–6F for example conformations). This is not surprising because conformational entropy of the glutamate side chain is expected to be higher than in aspartate, due to the additional carbon atom in the side chain, and, therefore, the existence of increased numbers of rotamers. Some typical trajectories for glutamate binding are shown in Fig 6G and 6H. Glutamate association occurred within the first 10 nanoseconds in all three subunits. However, stability in the binding pocket was decreased compared to the aspartate/Glt_*ph*_ complex, as indicated by several glutamate dissociation reactions observed in each subunit within the 100 ns simulation time. This result is in line with the reduced apparent affinity of the EAAT1/glutamate complex compared to Glt_*ph*_/aspartate [9, 13, 38, 39]. In addition, it indicates that the initial complex formation, before HP2 closure, is of low affinity, as had been suggested from rapid kinetic experiments and kinetic modeling [40].

## Discussion

In the structure of the archaeal glutamate transporter homologue Glt_*ph*_, the substrate, aspartate, is buried in the transport domain, closed off from the extracellular solution by HP2. Several charged conserved residues contribute to the binding of substrate (aspartate) [33, 34]. A highly conserved residue located in TM8, Arg-397, shows electrostatic binding interaction with the substrate β-carboxylate group. Mutational analysis based on a mammalian transporter provides evidence for this electrostatic interaction [33], i.e. neutralization of Arg-397 alters the type of amino acid transported from acidic to neutral amino acids [33, 41]. Other conserved residues close to the binding pocket predicted to contribute to substrate binding are Asp-394, Ala-358 from TM8, Thr-314 from TM7, Val-355 from HP2, Arg-276, and Ser-278 from HP1. Also, Gly-354 and Gly-357 located in HP2 work as a recognition group [33, 34, 42–44] for binding with substrate and forming the closed (occluded) state. In previous simulation studies [18], HP2 loop has been shown to undergo a large scale movement to accomplish the gating function of the binding pocket.

Here, we used three MD methods to accelerate the ligand binding process to a known transporter binding site. In all MD runs using accelerated protocols to keep the substrate within a user-defined cut-off distance from the binding site (7–12 Å), multiple binding and unbinding events were observed within 50 ns. In contrast, using purely conventional MD, spontaneous binding events did not occur when only three aspartate molecules were used in the simulation cell (one per subunit), or required 100s of ns of simulation time even when very high substrate concentrations were used (about 0.1 M). Therefore, the protocols applied here have the potential to significantly accelerate spontaneous binding events, while not using biasing force when the ligand is close to the binding site. As shown in the comparisons with the Glt_*ph*_ crystal structure, a near native binding mode is attained in the accelerated binding simulations, although the side chain of the bound ligand in the initial binding pose appears to have conformational flexibility (see below).

As expected, the simulation time needed to observe successful binding events depended on the cut-off distance, at which force application was switched off. We tested three cut-off distances, 7 to 12 Å, which yielded similar results with respect to the final binding pose, although the 12 Å cut-off required longer simulation times. However, even at 12 Å cut-off distance, binding was complete within 50 ns, indicating that the cut-off distance could even be increased, if required when applying these protocols to other targets.

Methods to observe ligand binding pathways in MD simulations have been applied in the past, in particular for G-protein coupled receptor ligand association [45, 46]. These simulations typically require micro-second time scales. A possibility to accelerate these simulations is to apply external forces, steering the ligand to the binding site. In an approach to observe spontaneous ligand binding, the supervised MD method was proposed [47]. Here, conventional MD simulations are stopped when the ligand moves away from the receptor binding site, and subsequently restarted. Thus, this method selects trajectories, in which the ligand continuously moves toward the receptor binding site. Binding events were observed in a 10s of nano-seconds time frame. Other approaches used funnel metadynamics [48], and methods based on electrostatic interactions [49]. Here, we combined user-defined force application with conventional MD when the ligand is close to the binding site, as well as preventing ligand diffusion through a flat-bottom harmonic potential, resulting in similar time savings, with a very simplified force application protocol. Flat-bottom harmonic potentials have been used previously in MD simulations of ligand binding to proteins and has been proven to accelerate the binding process.

The substrate binding and dissociation mechanism of glutamate transporters has been studied previously by using experimental approaches and several MD simulation methods. Bahar and colleagues were unable to observe spontaneous substrate binding when the substrate was placed outside of the "bowl" that is formed by the glutamate transporter trimeric assembly [18]. However, when substrate was placed manually within the bowl, spontaneous binding was observed, in analogy to our results using the accelerated substrate binding protocol. The interactions with Asp-390 were not noted in [18], possibly because the manual positioning of substrate in the bowl provided different starting positions than in our simulations, in which substrate was initially placed outside of the bowl.

In [27], substrate dissociation was studied at varying membrane potentials. The more negative the membrane potential, the faster the substrate was released to the aqueous solution. Together with results from [8, 18, 25, 50], it was concluded that a Na^+^ ion occupying the Na2 site acts as a lock to stabilize the substrate and close the HP2 loop, forming the occluded, substrate-bound state. To allow substrate dissociation, the Na^+^ ion needs to dissociate from the Na2 site first, which subsequently promotes opening of HP2, destabilizing the bound amino acid substrate. This mechanism was further supported by binding free energy calculations, showing that the Na2 position has relatively low affinity for Na^+^, 10–15 kcal/mol lower than the Na1 position [51, 52]. In our simulations, the Na2 site was not occupied, but Na1 site occupation was required for forming a stable complex with aspartate. Furthermore, It has been previously suggested that a highly conserved NMDGT motif [33, 34, 42, 53] located in TM7 contributes to substrate binding. In the TM8 helix, Arg-397 has a strong binding effect to the substrate through salt bridge interaction, as shown by mutagenesis studies [33]. Asp-390, Asp-394 (Glt_*ph*_ numbering) also have important roles and have been tested experimentally through mutagenesis in several glutamate transporter subtypes [33, 34]. Our MD simulation results are consistent with these previous studies, showing interaction of the substrate with these side chains before the stable, occluded substrate binding pose is reached. The results obtained from the accelerated MD simulations protocol were consistent with those from conventional MD, using high aspartate concentrations to achieve short lag periods for binding events. It should be noted that, in this work, creating high organic substrate concentration is a labor intensive, manual process, although scripts could be used to facilitate this process. Nevertheless, all three methods yielded similar results in terms of the final substrate binding pose and intermediates on the binding pathway. Furthermore, in all three methods, we observed that the Asp-390 and Asp-394 side chains could contribute to aspartate binding by forming an intermediate association state. The formation of these different binding positions may depend on the degree of binding pocket opening.

The importance of Arg-397 in controlling the conformation of aspartate was further highlighted in our results. Once the aspartate side chain carboxylate group formed the initial interaction with Arg-397, the *syn* conformation was observed, in which the aspartate carboxylates are on the same side of the C-C bond. However, after binding is complete, the *anti*-conformation of aspartate was stabilized, as demonstrated in the Glt_*ph*_ crystal structure. We evaluated the dihedral angle of the initial binding state as a function of time, as shown in S8 Fig. Bound aspartate spent about 70% of the time in the *anti*-conformation, and 30% time in the *syn-*conformation. This result suggests that the initial bound state is associated with considerable conformation flexibility of the aspartate side chain, while the final bound state with the substrate closed in by HP2 shows the much more pronounced stabilization of the *anti*-configuration (S9 Fig).

Finally, we investigated the trajectory of glutamate binding to the mammalian glutamate transporter EAAT1. The major conclusions, namely that initial, probably low-affinity binding precedes closure of HP2 and locking in of the substrate are similar compared to aspartate binding to Glt_*ph*_. In contrast, glutamate was less stable in the binding site than aspartate, due to the known lower affinity obtained from experiment, which is in the microM range. However, dissociation processes observed in the 100 ns time scale indicate short residency times, and, thus, much lower initial affinity. This is expected from experimental results from pre-steady-state kinetic studies, showing that the glutamate affinity increases from 100 μM upon initial binding, to the 5 μM range at steady state [40]. The likely structural interpretation is that the initial, low affinity binding state corresponds to the state observed here in the MD simulation approach, before HP2 closes. This initial state shows stability in the interactions of the α-carboxylate and amino group with the transporter, but the side chain remains mobile. Therefore, it can be speculated that the salt bridge of the side chain COO^-^ group with the arginine 459 (EAAT1 numbering) forms later, possibly aided by closure of hairpin loop 2.

In conclusion, we applied accelerated methods for MD simulations to study binding of ligands to binding sites, in particular for the glutamate transporter homologue Glt_*ph*_. The substrate (aspartate) spontaneous binding process was studied by applying a user-defined force protocol, while releasing any force when the substrate is within a user-defined distance from the binding site, as well as a method using a flat-bottom harmonic potential. Using these methods, the exact ligand binding process is largely unaffected by a biasing force. By applying these protocols, we largely save computational resources and time, and decrease the unnecessary simulation steps when the ligand is far away from the binding site. In the analysis of our simulation results, HP2 does not close in the timeframe of the simulations. In the TNa_1_Na_3_ bound state of the transporter, the HP2 loop opens within 5–10 ns of simulation, and once the gate is open the binding pocket becomes available for substrate to bind. The substrate initially binds with high conformational flexibility in the side chain, while upon closure of HP2 the *anti*-configuration is stabilized. Two potential modes of substrate association are illustrated by the simulations. After binding is complete the binding mode fits with the crystal structure binding mode (2nwx), and in an intermediate state, Asp-390 and Asp-394 located in TM7 contribute to binding the substrate. The results are also consistent with previous rports suggesting that the HP2 loop acts as a gate function to lock the substrate into the binding site, involving a conformational change slower than substrate binding. Finally, we extended the simulations to glutamate binding to EAAT1, a complex has not been investigated in structural studies. Similar to the aspartate/Glt_*ph*_ results, considerable conformational flexibility of the side chain was observed upon initial binding, most likely resulting in a low-affinity initial state. Overall, these results suggest intermediates on the substrate binding pathway that are not seen in the static crystal structures.

## Materials and methods

### Molecular dynamics simulations

The model system for MD simulations was generated with VMD software [54], and the Glt_ph_ (2nwx) structure was built by VMD. The Glt_ph_ structure was inserted into a pre-equilibrated POPC lipid bilayer with the dimensions of about 120 x 120 Å. TIP3P water was added to generate a box measuring about 100 Å in the z-direction. NaCl was added at a total concentration of 0.15 M and the system was neutralized. The total numbers of atoms in the Glt_ph_ system were 146757.

For the spontaneous aspartate binding simulations, we manually added 76 aspartates (about 0.1 M) evenly distributed extracellularly and intracellularly. Simulations were run using the CHARMM36 force field [30, 31] and CHARMM27 [32] including the CMAP dihedral corrections. NAMD [29] simulations were performed after 2000 steps minimization and 2 ns equilibration steps under constant pressure conditions (NPT), then used user-defined force in NAMD to run simulation as long as 40 ns, or switch to the ACEMD [55] program to run up to microsecond simulations to test spontaneous binding. The RMSD increased from 1.5 Å soon after the simulation began to ~3 Å after 2 ns of equilibration (S10 Fig), after which it was in steady state. In NAMD and ACEMD simulations, the cutoff for local electrostatic and van der Waals interactions was set to 12 Å. For long-range electrostatic interactions, we used the particle-mesh Ewald method implemented in NAMD. The time steps of the simulations were 2 fs. For ACEMD simulation, cut off for short-range interactions was 9 Å. The complete simulation system including substrate,2 Na (Na1, Na3 sites) or 1 Na (Na3 site) located at Na binding site, lipid and water is shown in Fig 1B.

### User-define force simulation

Simulations were initially started with aspartate located in the extracellular solution, we first removed sodium in the Na2 position in Glt_ph_ and performed 5 to 10 ns simulation using NAMD., the HP2 opened state subunit was selected to be reference and tracking aspartate binding distance.

We next selected the aspartate locations at two different distances from the target residue (27Å and 18Å) and started the force application and direction calculation steps. In the user-defined force simulation, we selected Arg-397 and atom name CG as reference atom, and selected aspartate atom name CA as force targeting atom, every two steps (or one step), user-defined force were applied to the target atom. Force direction was directly calculated by the vector between two atoms, and updated in every step of calculation. The force constant was 5 kcal/mol/Å^2^. The range selection and force direction calculation script are listed in S1 and S2 Figs.

### Application of a flat-bottom potential

Flat bottom potential application was used the same atom selection from *user-defined simulation*. Force constants were use 0.1 kcal/mol·Å. Cut-off distance use 12 Å in calculations. Relative energy as a function of the distance (Fig 3A) was plotted in Microsoft Excel.

### Trajectory analysis

The time evolution of distances and distribution function analysis were calculated by Tcl/Tk programs built in VMD [54] software. HP1-HP2 distance were calculated from atom CA in Ser-277 to atom CA in Pro-356. Substrate binding distance was calculated from atom CZ in Arg-397 to atom CA in aspartate.

The dihedral angle calculations were performed in VMD. Atom selection were from aspartate residue atom C, CA, CB, CG. All images were directly captured by VMD1.93 [54] and Pymol software.

## Supporting information

S1 Fig Principle of force application strategy. (PDF)

S2 Fig User-defined force calculation script. (PDF)

S3 Fig Hairpin loop 2 (HP2) opens within 100 ns of removing the substrate. (PDF)

S4 Fig Coordination of Glt_ph_-asp binding in intermediate state. (PDF)

S5 Fig Coordination in user-defined force simulation without Na1 occupied. (PDF)

S6 Fig Reduced stability of bound aspartate when the Na1 site is not occupied by Na^+^. (PDF)

S7 Fig Aspartate spontaneous binding process with 10 Å cut-off distance. (PDF)

S8 Fig Dihedral angle distribution for bound aspartate. (PDF)

S9 Fig Dihedral angle distribution from aspartate-bound state simulation with HP2 loop closed. (PDF)

S10 Fig Root Mean Square Deviation (RMSD) shows only minor difference between the two methods to accelerate substrate binding. (PDF)

## References

[1] Wadiche JI, Amara SG, Kavanaugh MP. Ion fluxes associated with excitatory amino acid transport. Neuron. 1995;15(3):721–8. Epub 1995/09/01. 10.1016/0896-6273(95)90159-0 . 7546750

[2] Zerangue N, Kavanaugh MP. Flux coupling in a neuronal glutamate transporter. Nature. 1996;383(6601):634–7. Epub 1996/10/17. 10.1038/383634a0 . 8857541

[3] Tanaka K, Watase K, Manabe T, Yamada K, Watanabe M, Takahashi K, et al. Epilepsy and exacerbation of brain injury in mice lacking the glutamate transporter GLT-1. Science. 1997;276(5319):1699–702. Epub 1997/06/13. 10.1126/science.276.5319.1699 . 9180080

[4] Masliah E, Alford M, DeTeresa R, Mallory M, Hansen L. Deficient glutamate transport is associated with neurodegeneration in Alzheimer’s disease. Ann Neurol. 1996;40(5):759–66. Epub 1996/11/01. 10.1002/ana.410400512 . 8957017

[5] Rothstein JD, Martin LJ, Kuncl RW. Decreased glutamate transport by the brain and spinal cord in amyotrophic lateral sclerosis. N Engl J Med. 1992;326(22):1464–8. Epub 1992/05/28. 10.1056/NEJM199205283262204 . 1349424

[6] Zerangue N, Kavanaugh MP. Interaction of L-cysteine with a human excitatory amino acid transporter. J Physiol. 1996;493 (Pt 2):419–23. Epub 1996/06/01. 10.1113/jphysiol.1996.sp021393 . 8782106 PMC1158927

[7] Reyes N, Ginter C, Boudker O. Transport mechanism of a bacterial homologue of glutamate transporters. Nature. 2009;462(7275):880–5. Epub 2009/11/20. 10.1038/nature08616 . 19924125 PMC2934767

[8] Boudker O, Ryan RM, Yernool D, Shimamoto K, Gouaux E. Coupling substrate and ion binding to extracellular gate of a sodium-dependent aspartate transporter. Nature. 2007;445(7126):387–93. Epub 2007/01/19. 10.1038/nature05455 . 17230192

[9] Canul-Tec JC, Assal R, Cirri E, Legrand P, Brier S, Chamot-Rooke J, et al. Structure and allosteric inhibition of excitatory amino acid transporter 1. Nature. 2017;544(7651):446–51. Epub 2017/04/21. 10.1038/nature22064 . 28424515 PMC5410168

[10] Kovermann P, Machtens JP, Ewers D, Fahlke C. A conserved aspartate determines pore properties of anion channels associated with excitatory amino acid transporter 4 (EAAT4). J Biol Chem. 2010;285(31):23676–86. Epub 2010/06/04. 10.1074/jbc.M110.126557 . 20519505 PMC2911312

[11] Silverstein N, Crisman TJ, Forrest LR, Kanner BI. Cysteine scanning mutagenesis of transmembrane helix 3 of a brain glutamate transporter reveals two conformationally sensitive positions. J Biol Chem. 2013;288(2):964–73. Epub 2012/11/29. 10.1074/jbc.M112.403576 . 23188832 PMC3543046

[12] Ruan Y, Miyagi A, Wang X, Chami M, Boudker O, Scheuring S. Direct visualization of glutamate transporter elevator mechanism by high-speed AFM. Proc Natl Acad Sci U S A. 2017;114(7):1584–8. Epub 2017/02/01. 10.1073/pnas.1616413114 . 28137870 PMC5320997

[13] Yernool D, Boudker O, Jin Y, Gouaux E. Structure of a glutamate transporter homologue from Pyrococcus horikoshii. Nature. 2004;431(7010):811–8. Epub 2004/10/16. 10.1038/nature03018 . 15483603

[14] Wang J, Albers T, Grewer C. Energy Landscape of the Substrate Translocation Equilibrium of Plasma-Membrane Glutamate Transporters. J Phys Chem B. 2018;122(1):28–39. Epub 2017/12/09. 10.1021/acs.jpcb.7b09059 . 29218993

[15] Silverstein N, Sliman A, Stockner T, Kanner BI. Both reentrant loops of the sodium-coupled glutamate transporters contain molecular determinants of cation selectivity. J Biol Chem. 2018;293(37):14200–9. Epub 2018/07/22. 10.1074/jbc.RA118.003261 . 30026234 PMC6139557

[16] Gu Y, Shrivastava IH, Amara SG, Bahar I. Molecular simulations elucidate the substrate translocation pathway in a glutamate transporter. Proc Natl Acad Sci U S A. 2009;106(8):2589–94. Epub 2009/02/10. 10.1073/pnas.0812299106 . 19202063 PMC2637273

[17] Zomot E, Bahar I. Intracellular gating in an inward-facing state of aspartate transporter Glt(Ph) is regulated by the movements of the helical hairpin HP2. J Biol Chem. 2013;288(12):8231–7. Epub 2013/02/07. 10.1074/jbc.M112.438432 . 23386619 PMC3605641

[18] Shrivastava IH, Jiang J, Amara SG, Bahar I. Time-resolved mechanism of extracellular gate opening and substrate binding in a glutamate transporter. J Biol Chem. 2008;283(42):28680–90. Epub 2008/08/06. 10.1074/jbc.M800889200 . 18678877 PMC2568915

[19] Guskov A, Jensen S, Faustino I, Marrink SJ, Slotboom DJ. Coupled binding mechanism of three sodium ions and aspartate in the glutamate transporter homologue GltTk. Nat Commun. 2016;7:13420. Epub 2016/11/11. 10.1038/ncomms13420 . 27830699 PMC5110648

[20] Ewers D, Becher T, Machtens JP, Weyand I, Fahlke C. Induced fit substrate binding to an archeal glutamate transporter homologue. Proc Natl Acad Sci U S A. 2013;110(30):12486–91. Epub 2013/07/11. 10.1073/pnas.1300772110 . 23840066 PMC3725095

[21] Hanelt I, Jensen S, Wunnicke D, Slotboom DJ. Low Affinity and Slow Na+ Binding Precedes High Affinity Aspartate Binding in the Secondary-active Transporter GltPh. J Biol Chem. 2015;290(26):15962–72. Epub 2015/04/30. 10.1074/jbc.M115.656876 . 25922069 PMC4481202

[22] Venkatesan S, Saha K, Sohail A, Sandtner W, Freissmuth M, Ecker GF, et al. Refinement of the Central Steps of Substrate Transport by the Aspartate Transporter GltPh: Elucidating the Role of the Na2 Sodium Binding Site. PLoS Comput Biol. 2015;11(10):e1004551. Epub 2015/10/21. 10.1371/journal.pcbi.1004551 . 26485255 PMC4618328

[23] Verdon G, Oh S, Serio RN, Boudker O. Coupled ion binding and structural transitions along the transport cycle of glutamate transporters. Elife. 2014;3:e02283. Epub 2014/05/21. 10.7554/eLife.02283 . 24842876 PMC4051121

[24] Machtens JP, Kortzak D, Lansche C, Leinenweber A, Kilian P, Begemann B, et al. Mechanisms of anion conduction by coupled glutamate transporters. Cell. 2015;160(3):542–53. Epub 2015/01/31. 10.1016/j.cell.2014.12.035 . 25635461

[25] Bastug T, Heinzelmann G, Kuyucak S, Salim M, Vandenberg RJ, Ryan RM. Position of the third Na+ site in the aspartate transporter GltPh and the human glutamate transporter, EAAT1. PLoS One. 2012;7(3):e33058. Epub 2012/03/20. 10.1371/journal.pone.0033058 . 22427946 PMC3302783

[26] Stolzenberg S, Khelashvili G, Weinstein H. Structural intermediates in a model of the substrate translocation path of the bacterial glutamate transporter homologue GltPh. J Phys Chem B. 2012;116(18):5372–83. Epub 2012/04/13. 10.1021/jp301726s . 22494242 PMC3350225

[27] Zielewicz L, Wang J, Ndaru E, Grewer CT. Transient Kinetics Reveal Mechanism and Voltage Dependence of Inhibitor and Substrate Binding to Glutamate Transporters. ACS Chem Biol. 2019. Epub 2019/04/27. 10.1021/acschembio.9b00194 . 31026143

[28] Grewer C, Watzke N, Wiessner M, Rauen T. Glutamate translocation of the neuronal glutamate transporter EAAC1 occurs within milliseconds. Proc Natl Acad Sci U S A. 2000;97(17):9706–11. Epub 2000/08/10. 10.1073/pnas.160170397 . 10931942 PMC16929

[29] Phillips JC, Braun R, Wang W, Gumbart J, Tajkhorshid E, Villa E, et al. Scalable molecular dynamics with NAMD. J Comput Chem. 2005;26(16):1781–802. Epub 2005/10/14. 10.1002/jcc.20289 . 16222654 PMC2486339

[30] Huang J, MacKerell AD Jr. CHARMM36 all-atom additive protein force field: validation based on comparison to NMR data. J Comput Chem. 2013;34(25):2135–45. Epub 2013/07/09. 10.1002/jcc.23354 . 23832629 PMC3800559

[31] Huang J, Rauscher S, Nawrocki G, Ran T, Feig M, de Groot BL, et al. CHARMM36m: an improved force field for folded and intrinsically disordered proteins. Nat Methods. 2017;14(1):71–3. Epub 2016/11/08. 10.1038/nmeth.4067 . 27819658 PMC5199616

[32] Mackerell AD Jr., Feig M, Brooks CL 3rd. Extending the treatment of backbone energetics in protein force fields: limitations of gas-phase quantum mechanics in reproducing protein conformational distributions in molecular dynamics simulations. J Comput Chem. 2004;25(11):1400–15. Epub 2004/06/09. 10.1002/jcc.20065 . 15185334

[33] Bendahan A, Armon A, Madani N, Kavanaugh MP, Kanner BI. Arginine 447 plays a pivotal role in substrate interactions in a neuronal glutamate transporter. J Biol Chem. 2000;275(48):37436–42. Epub 2000/09/09. 10.1074/jbc.M006536200 . 10978338

[34] Teichman S, Kanner BI. Aspartate-444 is essential for productive substrate interactions in a neuronal glutamate transporter. J Gen Physiol. 2007;129(6):527–39. Epub 2007/05/31. 10.1085/jgp.200609707 . 17535962 PMC2151622

[35] Tao Z, Gameiro A, Grewer C. Thallium ions can replace both sodium and potassium ions in the glutamate transporter excitatory amino acid carrier 1. Biochemistry. 2008;47(48):12923–30. Epub 2008/11/07. 10.1021/bi8017174 . 18986164 PMC2651767

[36] Grewer C, Zhang Z, Mwaura J, Albers T, Schwartz A, Gameiro A. Charge compensation mechanism of a Na+-coupled, secondary active glutamate transporter. J Biol Chem. 2012;287(32):26921–31. Epub 2012/06/19. 10.1074/jbc.M112.364059 . 22707712 PMC3411028

[37] Wang J, Zhang K, Goyal P, Grewer C. Mechanism and potential sites of potassium interaction with glutamate transporters. J Gen Physiol. 2020;152(10). Epub 2020/08/25. 10.1085/jgp.202012577 . 32835376 PMC7537348

[38] Wadiche JI, Kavanaugh MP. Macroscopic and microscopic properties of a cloned glutamate transporter/chloride channel. J Neurosci. 1998;18(19):7650–61. Epub 1998/09/19. 10.1523/JNEUROSCI.18-19-07650.1998 . 9742136 PMC6793006

[39] Arriza JL, Fairman WA, Wadiche JI, Murdoch GH, Kavanaugh MP, Amara SG. Functional comparisons of three glutamate transporter subtypes cloned from human motor cortex. J Neurosci. 1994;14(9):5559–69. Epub 1994/09/01. 10.1523/JNEUROSCI.14-09-05559.1994 . 7521911 PMC6577102

[40] Grewer C, Rauen T. Electrogenic glutamate transporters in the CNS: molecular mechanism, pre-steady-state kinetics, and their impact on synaptic signaling. J Membr Biol. 2005;203(1):1–20. Epub 2005/04/19. 10.1007/s00232-004-0731-6 . 15834685 PMC2389879

[41] Grewer C, Balani P, Weidenfeller C, Bartusel T, Tao Z, Rauen T. Individual subunits of the glutamate transporter EAAC1 homotrimer function independently of each other. Biochemistry. 2005;44(35):11913–23. Epub 2005/09/01. 10.1021/bi050987n . 16128593 PMC2459315

[42] Seal RP, Leighton BH, Amara SG. A model for the topology of excitatory amino acid transporters determined by the extracellular accessibility of substituted cysteines. Neuron. 2000;25(3):695–706. Epub 2000/04/25. 10.1016/s0896-6273(00)81071-5 . 10774736

[43] Tao Z, Zhang Z, Grewer C. Neutralization of the aspartic acid residue Asp-367, but not Asp-454, inhibits binding of Na+ to the glutamate-free form and cycling of the glutamate transporter EAAC1. J Biol Chem. 2006;281(15):10263–72. Epub 2006/02/16. 10.1074/jbc.M510739200 . 16478724 PMC2430067

[44] Teichman S, Qu S, Kanner BI. Conserved asparagine residue located in binding pocket controls cation selectivity and substrate interactions in neuronal glutamate transporter. J Biol Chem. 2012;287(21):17198–205. Epub 2012/04/12. 10.1074/jbc.M112.355040 . 22493292 PMC3366809

[45] Dror RO, Pan AC, Arlow DH, Borhani DW, Maragakis P, Shan Y, et al. Pathway and mechanism of drug binding to G-protein-coupled receptors. Proc Natl Acad Sci U S A. 2011;108(32):13118–23. Epub 2011/07/23. 10.1073/pnas.1104614108 . 21778406 PMC3156183

[46] Dror RO, Green HF, Valant C, Borhani DW, Valcourt JR, Pan AC, et al. Structural basis for modulation of a G-protein-coupled receptor by allosteric drugs. Nature. 2013;503(7475):295–9. Epub 2013/10/15. 10.1038/nature12595 . 24121438

[47] Sabbadin D, Moro S. Supervised molecular dynamics (SuMD) as a helpful tool to depict GPCR-ligand recognition pathway in a nanosecond time scale. J Chem Inf Model. 2014;54(2):372–6. Epub 2014/01/25. 10.1021/ci400766b . 24456045

[48] Limongelli V, Bonomi M, Parrinello M. Funnel metadynamics as accurate binding free-energy method. Proc Natl Acad Sci U S A. 2013;110(16):6358–63. Epub 2013/04/05. 10.1073/pnas.1303186110 . 23553839 PMC3631651

[49] Spitaleri A, Decherchi S, Cavalli A, Rocchia W. Fast Dynamic Docking Guided by Adaptive Electrostatic Bias: The MD-Binding Approach. J Chem Theory Comput. 2018;14(3):1727–36. Epub 2018/01/20. 10.1021/acs.jctc.7b01088 . 29351374

[50] Huang Z, Tajkhorshid E. Dynamics of the extracellular gate and ion-substrate coupling in the glutamate transporter. Biophys J. 2008;95(5):2292–300. Epub 2008/06/03. 10.1529/biophysj.108.133421 . 18515371 PMC2517027

[51] Setiadi J, Heinzelmann G, Kuyucak S. Computational Studies of Glutamate Transporters. Biomolecules. 2015;5(4):3067–86. Epub 2015/11/17. 10.3390/biom5043067 . 26569328 PMC4693270

[52] Heinzelmann G, Bastug T, Kuyucak S. Mechanism and energetics of ligand release in the aspartate transporter GltPh. J Phys Chem B. 2013;117(18):5486–96. Epub 2013/04/18. 10.1021/jp4010423 . 23590433

[53] Rosental N, Bendahan A, Kanner BI. Multiple consequences of mutating two conserved beta-bridge forming residues in the translocation cycle of a neuronal glutamate transporter. J Biol Chem. 2006;281(38):27905–15. Epub 2006/07/28. 10.1074/jbc.M600331200 . 16870620

[54] Humphrey W, Dalke A, Schulten K. VMD: visual molecular dynamics. J Mol Graph. 1996;14(1):33–8, 27–8. Epub 1996/02/01. 10.1016/0263-7855(96)00018-5 . 8744570

[55] Harvey MJ, Giupponi G, Fabritiis GD. ACEMD: Accelerating Biomolecular Dynamics in the Microsecond Time Scale. J Chem Theory Comput. 2009;5(6):1632–9. Epub 2009/06/09. 10.1021/ct9000685 . 26609855

