# Supplementary material for: Observing spontaneous, accelerated substrate binding in molecular dynamics simulations of glutamate transporters
Source: PLoS One. 2021 Apr 23;16(4):e0250635. doi: 10.1371/journal.pone.0250635 (PMC8064580; doi:10.1371/journal.pone.0250635)
Supplement: S1 Fig — (PDF) [file pone.0250635.s001.pdf]

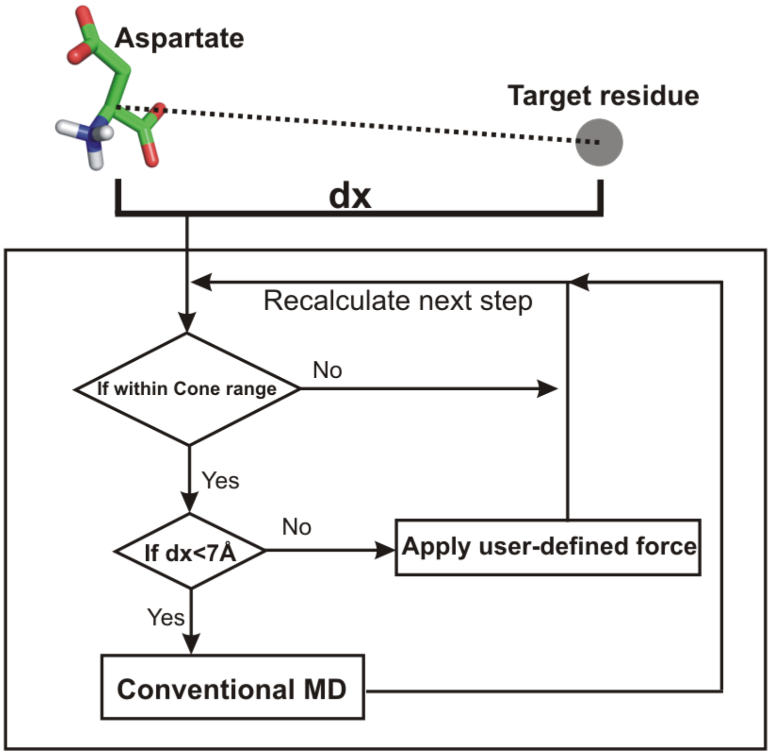

Fig. S1 principle of force application strategy

Flow control scheme of the calculation is indicated by arrows. The cone region (Fig. 1C) was used as the first decision criterion and the distance dx 7Å or 12Å from the target residue to define the boundary was the second decision criterion. Direction and distance of aspartate were calculated in every step.
