# Supplementary material for: Observing spontaneous, accelerated substrate binding in molecular dynamics simulations of glutamate transporters
Source: PLoS One. 2021 Apr 23;16(4):e0250635. doi: 10.1371/journal.pone.0250635 (PMC8064580; doi:10.1371/journal.pone.0250635)
Supplement: S2 Fig — (PDF) [file pone.0250635.s002.pdf]

```
#correct scale of force direction#
set a [expr $k*($r2x-$r1x)]
set b [expr $k*($r2y-$r1y)]
set c [expr $k*($r2z-$r1z)]
lappend direction $a $b $c
#define region#
set sxy [expr $r1x*$r1x + $r1y*$r1y]
set sz [expr ($r1z+15)*($r1z+15)]
#addforce or not#
set result [veclength [vecsub $r1 $r2]]
print "Distance is $result"
print "max distance is $max"
if {$sxy <= $sz && $r1z>0} {
    print "target atom within cone range"
    if {$result >= $max} {
        print "result >= $max, too far push close"
        addforce $a1 $direction
    }
}
```

Fig. S2 User-defined force calculation script

User-defined force calculation script were corresponded with flow control scheme in Fig.S1. it included the range selection and decision steps.
