# Supplementary material for: Observing spontaneous, accelerated substrate binding in molecular dynamics simulations of glutamate transporters
Source: PLoS One. 2021 Apr 23;16(4):e0250635. doi: 10.1371/journal.pone.0250635 (PMC8064580; doi:10.1371/journal.pone.0250635)
Supplement: S3 Fig — (PDF) [file pone.0250635.s003.pdf]

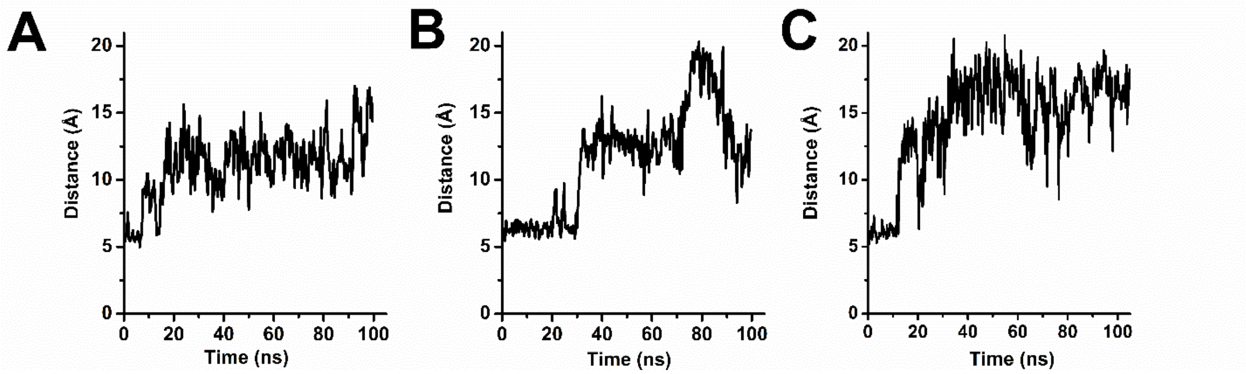

Fig. S3: Hairpin loop 2 (HP2) opens within 100 ns of removing the substrate

The distance between the tips of HP1 and HP2 as a function of time for three representative simulations after aspartate was removed from the binding site. Atom selection and calculation methods were same as in Fig. 2.
