# Supplementary material for: Observing spontaneous, accelerated substrate binding in molecular dynamics simulations of glutamate transporters
Source: PLoS One. 2021 Apr 23;16(4):e0250635. doi: 10.1371/journal.pone.0250635 (PMC8064580; doi:10.1371/journal.pone.0250635)
Supplement: S4 Fig — (PDF) [file pone.0250635.s004.pdf]

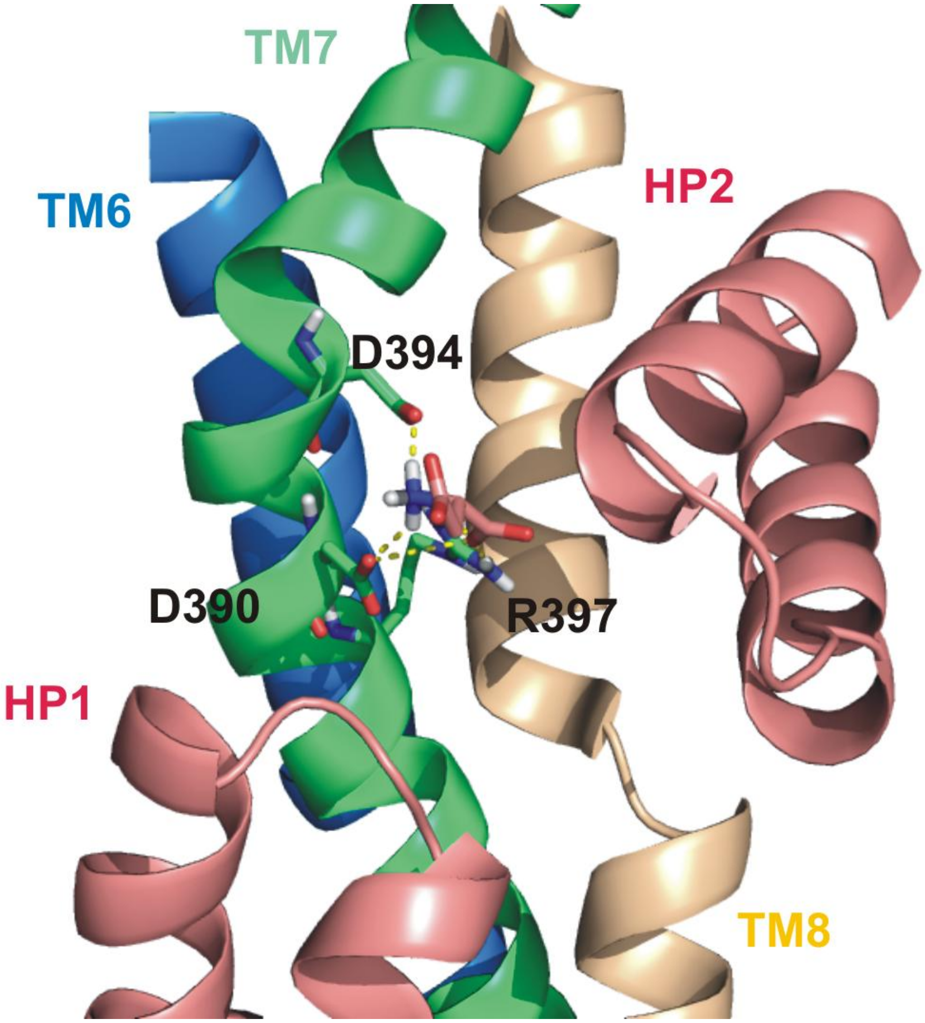

Fig. S4: Coordination of Glt$_{ph}$-asp binding in intermediate state

This intermediate state was short lived and above the original binding pocket. The side chains of Arg397, Asp-394 and Asp-390 from the same subunit contribute to substrate stabilization.
