# Supplementary material for: Observing spontaneous, accelerated substrate binding in molecular dynamics simulations of glutamate transporters
Source: PLoS One. 2021 Apr 23;16(4):e0250635. doi: 10.1371/journal.pone.0250635 (PMC8064580; doi:10.1371/journal.pone.0250635)
Supplement: S5 Fig — (PDF) [file pone.0250635.s005.pdf]

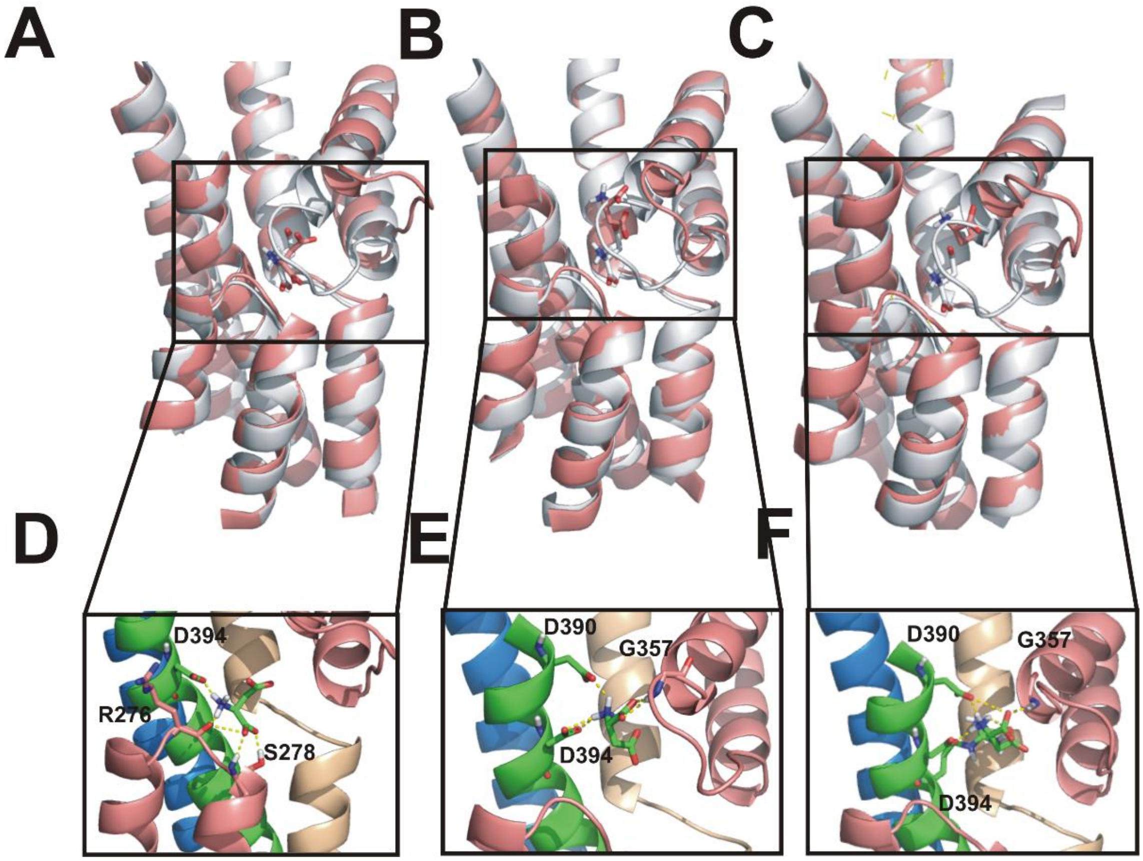

Fig. S5: Coordination in user-defined force simulation without Na1 occupied

Illustration of substrate-protein binding steps. In a representative simulation with user-defined force, within a short period of simulation time, aspartate will move close to the HP2 loop, but the binding pocket is not accessible when the HP2 loop is not fully open. Once the HP2-open state forms (HP2-HP1 distance larger than 10Å), aspartate is allowed to move inside the binding pocket. (A-C) Coordination state with open loop from simulation results is shown in pink. Comparison with the crystal structure (2nwx) is shown in grey. All simulation results were from sampling by adding user-defined force to conventional MD simulations. Two distinguishable binding positions are shown in (A) and (B-C). One overlaps with the binding site in the crystal structure (D) and another (E, F) is located at an elevated Z-axis position. (E) Aspartate was in HP2 half-open state, and (F) in HP2 full-open state.
