# Supplementary material for: Observing spontaneous, accelerated substrate binding in molecular dynamics simulations of glutamate transporters
Source: PLoS One. 2021 Apr 23;16(4):e0250635. doi: 10.1371/journal.pone.0250635 (PMC8064580; doi:10.1371/journal.pone.0250635)
Supplement: S6 Fig — (PDF) [file pone.0250635.s006.pdf]

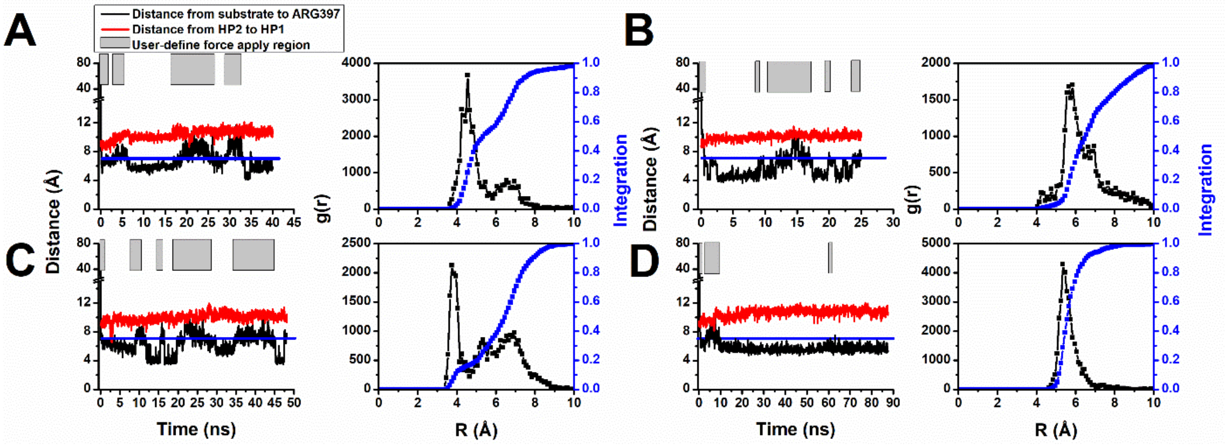

Fig. S6: Reduced stability of bound aspartate when the Na1 site is not occupied by Na^+

Four independent simulations were performed at conditions when the Na1 site was not not occupied. Time evolution of distance from the binding site reference residue (black) and HP2-HP1 distance (red) are shown in each panel, respectively. The blue line indicates the user-defined force boundary and the grey bar region indicates the time of force application,

The right panels show the aspartate radial distribution function (black) and cumulative radial distribution function (blue). Results (A) to (D) were analyzed with same method as in Fig. 4 and Fig. 6. Aspartate populated distance are from 4 to 10 Å to Arg-397.
