# Supplementary material for: Observing spontaneous, accelerated substrate binding in molecular dynamics simulations of glutamate transporters
Source: PLoS One. 2021 Apr 23;16(4):e0250635. doi: 10.1371/journal.pone.0250635 (PMC8064580; doi:10.1371/journal.pone.0250635)
Supplement: S7 Fig — (PDF) [file pone.0250635.s007.pdf]

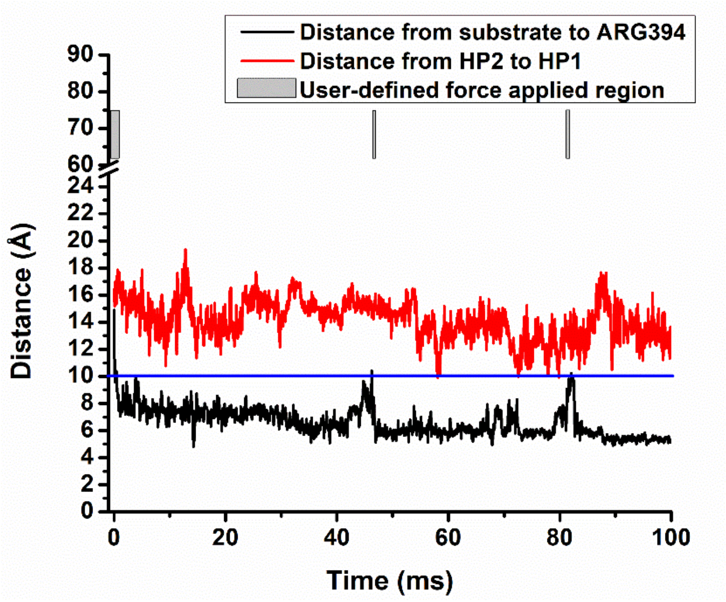

Fig. S7: Aspartate spontaneous binding process with 10 Å cut-off distance

Simulations were performed under same conditions as in Fig. 2 but with 10 Å cut-off distance.
