# Supplementary material for: Observing spontaneous, accelerated substrate binding in molecular dynamics simulations of glutamate transporters
Source: PLoS One. 2021 Apr 23;16(4):e0250635. doi: 10.1371/journal.pone.0250635 (PMC8064580; doi:10.1371/journal.pone.0250635)
Supplement: S8 Fig — (PDF) [file pone.0250635.s008.pdf]

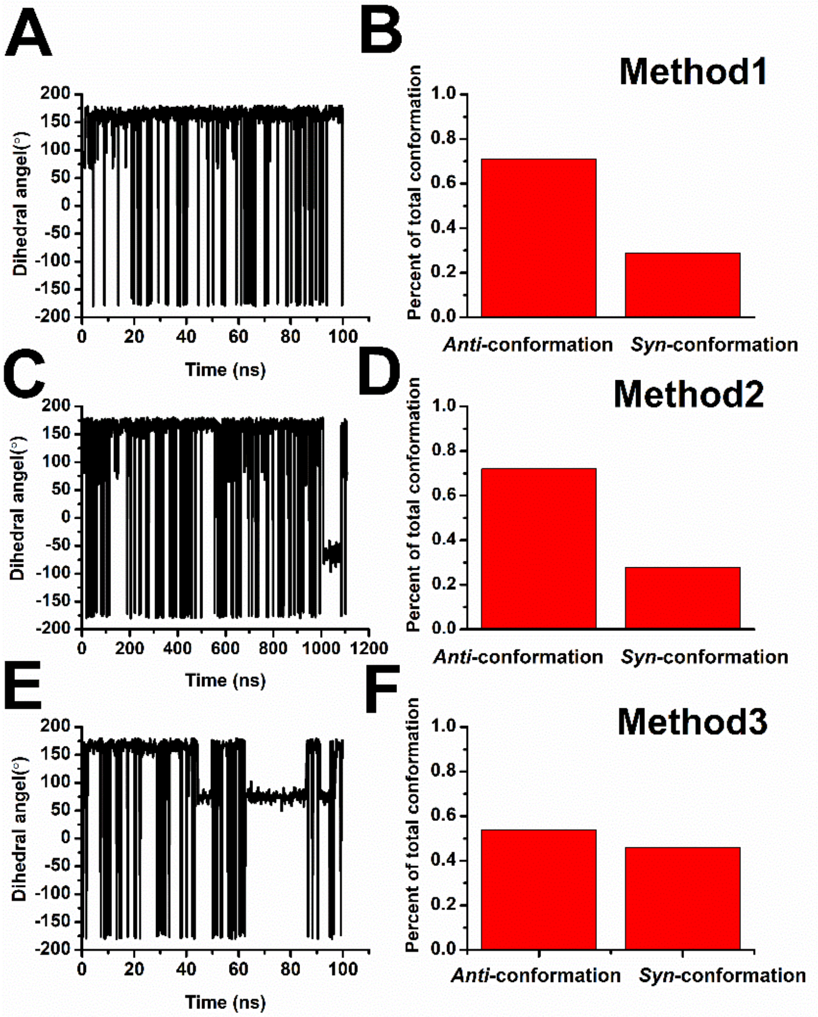

Fig. S8 Dihedral angle distribution for bound aspartate

Dihedral angles were calculated and selected from three different methods and shown from (A) to (F). The calculations were selected from aspartate residue atoms C, CA, CB, CG. Dihedral angels were plotted as a function of simulation time in (A)(C)(D). Distribution of *anti*-, *syn*-conformations were calculated in (B)(D)(F).
