# Supplementary material for: Observing spontaneous, accelerated substrate binding in molecular dynamics simulations of glutamate transporters
Source: PLoS One. 2021 Apr 23;16(4):e0250635. doi: 10.1371/journal.pone.0250635 (PMC8064580; doi:10.1371/journal.pone.0250635)
Supplement: S9 Fig — (PDF) [file pone.0250635.s009.pdf]

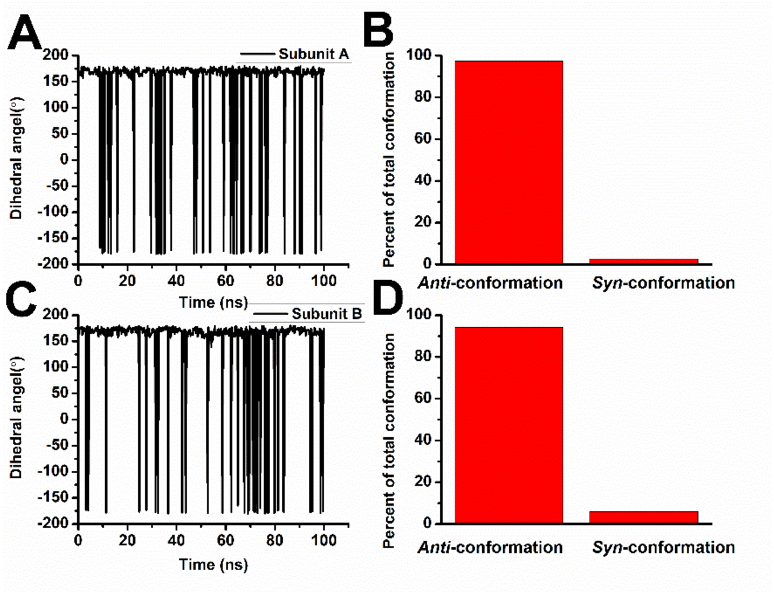

Fig. S9: Dihedral angle distribution from aspartate-bound state simulation with HP2 loop closed

Dihedral angles were calculated from a 100 ns simulation in the aspartate-bound state with HP2 loop closed. Distribution (A) and (C) were plotted using the same method in Fig.S7. Distributions of *anti*-, *syn*-conformation were calculated in (B)(D).
