# Supplementary material for: Observing spontaneous, accelerated substrate binding in molecular dynamics simulations of glutamate transporters
Source: PLoS One. 2021 Apr 23;16(4):e0250635. doi: 10.1371/journal.pone.0250635 (PMC8064580; doi:10.1371/journal.pone.0250635)
Supplement: S10 Fig — (PDF) [file pone.0250635.s010.pdf]

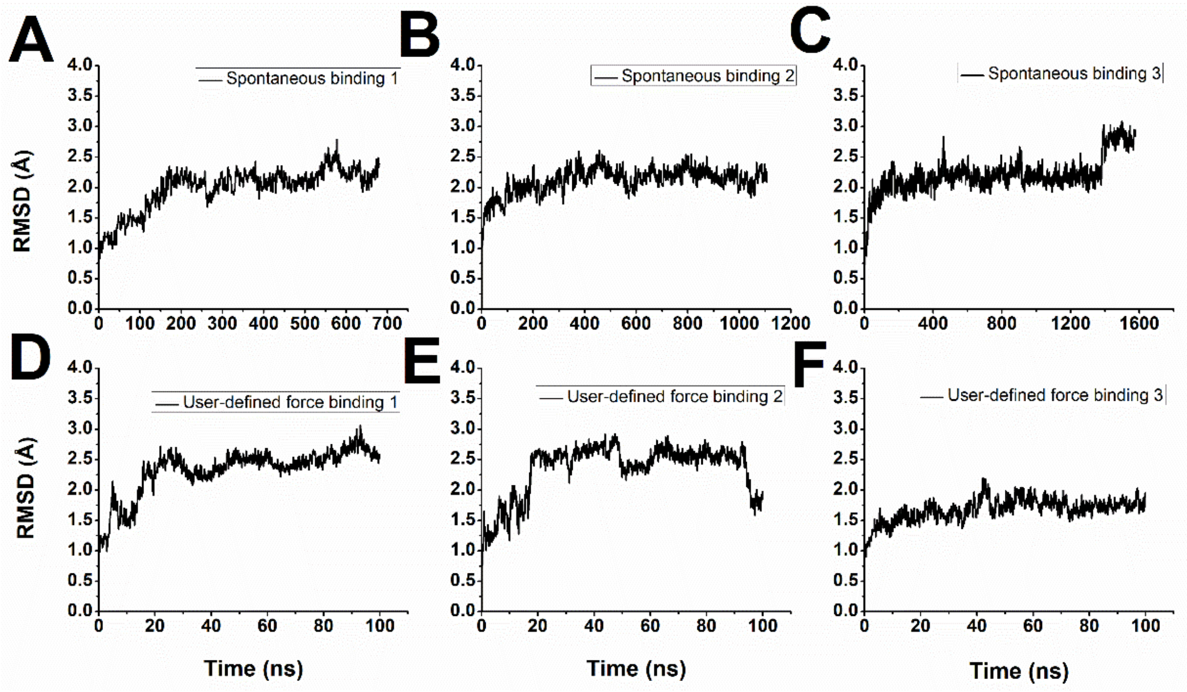

Fig. S10: Root mean square deviation (RMSD) shows only minor difference between the two methods to accelerate substrate binding

RMSD calculations were calculated in a specific subunit, in which substrate binding took place. Backbone atom (CA) were selected to do the analysis from their initial coordinates. (A) to (C) are spontaneous binding results, using high aspartate concentration, (D) to (F) are results from user-defined force binding simulations.
